# Understanding the physiological and biological response to ambient heat exposure in pregnancy: protocol for a systematic review and meta-analysis

**DOI:** 10.1136/bmjopen-2024-085314

**Published:** 2024-07-05

**Authors:** Ana Bonell, Leonidas G Ioannou, Jane Elizabeth Hirst, Andreas Flouris

**Affiliations:** 1 Medical Research Council Unit The Gambia, London School of Hygiene & Tropical Medicine, London, UK; 2 FAME Laboratory, Department of Physical Education and Sport Science, University of Thessaly, Volos, Greece; 3 The George Institute for Global Health, Imperial College London, London, UK; 4 Nuffield Department of Women's & Reproductive Health, University of Oxford, Oxford, UK

**Keywords:** Pregnant Women, Physiology, Climate Change, Fetal medicine

## Abstract

**Introduction:**

Climate change increases not only the frequency, intensity and duration of extreme heat events but also annual temperatures globally, resulting in many negative health effects, including harmful effects on pregnancy and pregnancy outcomes. As temperatures continue to increase precipitously, there is a growing need to understand the underlying biological pathways of this association. This systematic review will focus on maternal, placental and fetal changes that occur in pregnancy due to environmental heat stress exposure, in order to identify the evidence-based pathways that play a role in this association.

**Methods and analysis:**

We will follow the Preferred Reporting Items for Systematic Reviews and Meta-Analyses guidelines. We will search PubMed and Ovid Embase databases from inception using tested and validated search algorithms. Inclusion of any studies that involve pregnant women and have measured environmental heat stress exposure and either maternal, placental or fetal physiological or biochemical changes and are available in English. Modelling studies or those with only animals will be excluded. The risk of bias will be assessed using the Office of Health Assessment and Translation tool. Abstract screening, data extraction and risk of bias assessment will be conducted by two independent reviewers.

Environmental parameters will be reported for each study and where possible these will be combined to calculate a heat stress indicator to allow comparison of exposure between studies. A narrative synthesis will be presented following standard guidelines. Where outcome measures have at least two levels of exposure, we will conduct a dose–response meta-analysis should there be at least three studies with the same outcome. A random effects meta-analysis will be conducted where at least three studies give the same outcome.

**Ethics and dissemination:**

This systematic review and meta-analysis does not require ethical approval. Dissemination will be through peer-reviewed journal publication and presentation at international conferences/interest groups.

**PROSPERO registration number:**

CRD42024511153.

**STRENGTHS AND LIMITATIONS OF THIS STUDY**

- This systematic review will summarise evidence-based biological changes that occur when pregnancy women are exposure to environmental heat stress on maternal, placental and fetal physiology and biochemistry.
- The evidence synthesis and meta-analysis will be useful in ongoing work to develop evidence-based solutions and interventions in this field.
- The small number of studies, dissimilar methodology and variation in exposure may prevent quantitative synthesis in all outcomes of interest.

## Introduction

### Background

Anthropogenic climate change has led to annual global temperatures rising rapidly.^1 2^ With this, there is an increase in the frequency, duration and intensity of extreme heat events.^3–5^ Extreme heat has been shown to be the deadliest of extreme weather events with projections, indicating that this is likely to worsen in the coming years.^6 7^

Pregnant women have been identified as a vulnerable group to the health impacts of extreme heat. In particular, maternal heat exposure is associated with an increased risk of both maternal morbidity and adverse pregnancy outcomes.^8^ Despite multiple studies showing that extreme heat is associated with poor birth outcomes,^9 10^ data on the underlying pathophysiological mechanisms remain sparse.^11^ As work on effective and evidence-based interventions to reduce the health impacts of extreme heat develops at pace, this systematic review summarises our underlying knowledge of the impact of heat on maternal, placental and fetal physiology and highlights both knowledge gaps and potential pathways that could be considered in intervention studies.

### Description of exposure: environmental heat stress

Humans experience external thermal stress from their surroundings and environmental conditions and internal thermal stress from metabolic heat production.^12^ Core temperature is kept within a close range around 37°C to ensure vital intracellular and extracellular functions perform optimally. In pregnancy, there are the additional considerations of fetal thermal generation and loss, and the health implications of thermal stress on both mother and fetus.^13 14^ This heat balance is maintained by behavioural and physiological changes, both conscious and unconscious. Some behavioural changes include removing of clothing layers, seeking shade or air conditioning, resting and water submersion to name a few.^15^ The physiological responses include sweating, peripheral vasodilation and reduced metabolic heat production.^12^ Should these mechanisms be overwhelmed, then the core body temperature rises and adverse health effects are experienced, including heat illness, heat stroke and in pregnancy both maternal morbidity and adverse fetal outcomes.^8 9 16^

External conditions can be defined by temperature alone, or by heat stress indices such as the Wet Bulb Globe Temperature or the Universal Thermal Climate Index. These heat stress indices attempt to protect human health by preventing adverse health outcomes by modelling the physiological response, and/or using some combination of humidity, solar radiation and wind speed with air temperature to give a single overall estimate of heat stress exposure.^17^ With the ongoing climate crisis, populations around the world are increasingly exposed to extreme heat and extreme heat stress, which threatens the gains that have been made in maternal and neonatal mortality in recent years.^18^ It is critical to understand the mechanisms by which heat exposure increases maternal morbidity and adverse pregnancy outcomes.

### Description of outcome

Maternal exposure to high ambient conditions has been shown to increase the risks of early pregnancy loss,^19^ congenital abnormalities,^20^ preterm birth,^21^ low birth weight,^22 23^ small-for-gestational age,^24^ pre-eclampsia,^25^ gestational diabetes^26^ and stillbirth.^27^ This is likely through various biological and physiological pathways although extensive literature on this is lacking.^11^ Based on findings from animal studies, the absolute threshold of effect for teratogenicity of maternal core temperature depends on several factors: gestational age at exposure, length of exposure and species-specific factors.^28^ It is thought likely that the most common adverse outcome of hyperthermia in early pregnancy is fetal loss, however, if the exposure is later in the first trimester exposure, then congenital abnormalities are common, especially in the central nervous system.^14^ However, in the case of environmental heat stress exposure and effect, it is likely that physiological adaptation to maintain maternal core temperature may adversely affect the fetus. Therefore, the rise in maternal core temperature is likely only one of several pathways causing adverse outcomes. Key pathways that will be explored in this systematic review include: (1) evidence of maternal heat strain defined by changes in maternal physiology, activation of the maternal inflammatory system, changes in maternal heat shock protein (HSP) expression and alteration in oxytocin and other hormonal levels, (2) evidence of placental heat strain defined by changes in placental blood flow, changes in placental epigenetic expression and changes in circulating placental biomarkers, (3) evidence of fetal heat strain defined by changes in fetal physiology, fetal heart rate and fetal movements.

### Rationale for the systematic review

With ongoing global exposure to high and ever-increasing temperatures, understanding the health implications of heat has become very important. Many studies and several systematic reviews have demonstrated the clear link between heat exposure and adverse pregnancy outcomes, but a systematic review of the mechanistic pathways is missing. This is an urgent knowledge gap as there is a need to adapt to the current climate and find evidence-based and effective solutions. Understanding the physiological pathways in more detail will help to inform which interventions may be effective, identify which groups are most vulnerable to the effects of heat and inform the evaluation of these potentially effective interventions.

### Objectives

In this paper, we describe the protocol for a systematic review that aims to expand our understanding on the relationship between environmental heat stress exposure and maternal, placental and fetal physiological and biological responses, using the Population/Exposure/Comparator/Outcome/Study design.

### Research question

What is the effect of an increased environmental heat stress exposure (E) on maternal thermophysiology (heart rate, stroke volume, cardiac output, skin temperature, core body temperature, uterine blood flow, hydration state, vasomotor tone, sudomotor tone) and comfort (thermal comfort, thermal sensation, perceived exertion), maternal circulating biomarkers (inflammatory markers, HSP, hormones and plasma glucose level), the placenta (placental epigenetic expression, placental vasculature, placental HSP expression, placental extracellular vesicles) and fetal physiology (umbilical artery and other fetal measures of blood flow, fetal heart rate, fetal movements, uterine contractions) (O) compared with low or no environmental heat stress (C) in pregnant women (P) within observational and experimental studies (S)?

## Methods and analysis

We will follow the Preferred Reporting Items for Systematic Reviews and Meta-Analyses (PRISMA) guidelines. The protocol has been registered in the International Prospective Register of Systematic Reviews.^29^ Studies will be included if they fulfil all of the following criteria.

### Eligibility criteria

1. Study declares to be evaluating maternal, placental or fetal exposure to heat.
2. The source of the exposure is clearly defined (eg, ambient heat, solar radiation, humidity, wind, clothing, metabolic rate, thermal stress indicator, etc).
3. Any of the following outcomes were evaluated: heart rate, stroke volume, cardiac output, skin temperature, core body temperature, uterine blood flow, hydration state, vasomotor tone, sudomotor tone, thermal comfort, thermal sensation, perceived exertion, maternal inflammatory markers, HSP, hormones, plasma glucose, placental epigenetic expression, placental vasculature, placental HSP expression, placental extracellular vesicles, umbilical artery and other fetal measures of blood flow, fetal heart rate, fetal movements, uterine contractions.
4. The experimental design has been granted ethics approval from the relevant authority or formal waiver.
5. The study has undergone peer review.
6. English language.
7. No date restrictions.

### Types of population

We will include only human pregnancy data since animal studies are limited by anatomical differences, litter sizes, sweating function and thermal thresholds. We will include pregnant women exposed to environmental heat stress in either a laboratory or field settings, but we will not include fever studies, where changes in core maternal temperature are due to infectious causes, as these involve different pathophysiological pathways.

### Types of exposure

We will include environmental heat stress exposure from external conditions (eg, high air temperature) in both heat chamber/laboratory studies and field-based studies. Metabolic heat production from physical activity will be included if this is an aspect of the study. Heat illness can occur from both or either of these heat exposures and, therefore it is important to include them in our understanding of the pathways.

The environmental parameters reported in each study, including air temperature, humidity, wind and radiant heat, will be used to calculate a heat stress indicator (eg, wet-bulb globe temperature). This will provide a more comprehensive understanding of the thermal conditions experienced by participants in each study. Additionally, this indicator will enable us to establish a common denominator for generating dose–response relationships. Multiple indicators will be calculated, and those that prove to be most effective will be incorporated into our analyses.

### Inclusion criteria

Studies will be included if they involve pregnant women and have measured the defined exposure of environmental heat stress and one of the predefined outcomes of maternal, placental or fetal physiological or biochemical changes. We will include the following study design: randomised and non-randomised controlled trials, with comparison groups or crossover design; observational studies with individuals exposed to different conditions including cohort studies, cross-sectional and case–control studies.

### Exclusion criteria

We will exclude environmental epidemiology studies of heat and birth outcome where no physiological measurements are included. We will also exclude modelling studies where human participants are not included, or animal studies or animal models that are extrapolated to human estimates. We will also exclude review papers where no original data are collected.

### Information sources and search strategy

We will perform a systematic search of PubMed and Ovid Embase databases using tested and validated search algorithms. As there are no systematic reviews on this exact topic, a list of seven gold-standard papers was created by an expert in maternal physiology (AB), while the search algorithm was independently developed by another researcher (LGI). The validity of the algorithm was confirmed by ensuring that all seven gold-standard papers were identified by the search algorithm (see online supplemental file 1). Table 1 gives the search algorithm for PubMed (see online supplemental file 1 for the search algorithms for Ovid Embase). We will also complement our search strategy by searching the reference lists of the included studies and the reference list of any relevant systematic or narrative reviews. Grey literature, such as theses, conference proceedings and reports will also be searched to minimise the risk of publication bias.

10.1136/bmjopen-2024-085314.supp1 Supplementary data

**Table 1:** Search algorithm for PubMed, validated against identification of the gold-standard list

#	Search algorithm
1	(heat*(Title/Abstract)AND (stress(Title/Abstract)OR strain(Title/Abstract)))
2	(hot(Title/Abstract)AND (temperature*(Title/Abstract)OR condition*(Title/Abstract)OR environment*(Title/Abstract)OR workplace*(Title/Abstract)OR ambient*(Title/Abstract)))
3	weather*(Title/Abstract)OR atmospher*(Title/Abstract)OR meteorolog*(Title/Abstract)
4	#1 OR #2 OR #3
5	(maternal*(Title/Abstract)OR mother*(Title/Abstract)OR pregnan*(Title/Abstract)OR woman(Title/Abstract)OR women(Title/Abstract))
6	physiolog*(Title/Abstract)
7	heart rate*(Title/Abstract)
8	stroke volume*(Title/Abstract)
9	cardiac output*(Title/Abstract)
10	((skin*(Title/Abstract)OR core*(Title/Abstract)OR body(Title/Abstract)OR bodies(Title/Abstract)) AND (temperature*(Title/Abstract)))
11	(uterine*(Title/Abstract)AND blood flow*(Title/Abstract))
12	(hydrat*(Title/Abstract)OR euhydrat*(Title/Abstract)OR dehydrat*(Title/Abstract)OR hypohydrat*(Title/Abstract))
13	(vasomot*(Title/Abstract)OR vasoconstrict*(Title/Abstract)OR vasodilat*(Title/Abstract))
14	(sudomotor(Title/Abstract)OR sweat*(Title/Abstract))
15	(thermal(Title/Abstract)AND (comfort*(Title/Abstract)OR sensation*(Title/Abstract)))
16	(exertion(Title/Abstract)OR fatigue*(Title/Abstract)OR tired*(Title/Abstract))
17	(inflammat*(Title/Abstract)AND marker*(Title/Abstract))
18	heat shock protein*(Title/Abstract)
19	hormon*(Title/Abstract)
20	plasma(Title/Abstract)AND glucose(Title/Abstract)
21	(placenta*(Title/Abstract)AND (epigenetic expression*(Title/Abstract)OR vasculature*(Title/Abstract)OR heat shock protein*(Title/Abstract)OR extracellular vesicle*(Title/Abstract)OR weight*(Title/Abstract)OR volume*(Title/Abstract)OR DNA methylation*(Title/Abstract)))
22	umbilical(Title/Abstract)AND arter*(Title/Abstract)
23	((fetus*(Title/Abstract)OR fetal(Title/Abstract)OR antenatal(Title/Abstract)OR embryonic(Title/Abstract)OR prenatal(Title/Abstract)OR unborn(Title/Abstract)) AND (heart rate*(Title/Abstract)OR movement*(Title/Abstract)OR growth restriction*(Title/Abstract)OR (uterine(Title/Abstract)AND contraction*(Title/Abstract))))
24	#6 OR #7 OR #8 OR #9 OR #10 OR #11 OR #12 OR #13 OR #14 OR #15 OR #16 OR #17 OR #18 OR #19 OR #20 OR #21 OR #22 OR #23
25	#4 AND #5 AND #24
26	#25 NOT (animals(mh] NOT humans(mh))

### Study selection

Publications from the combined searches will be imported to Endnote and duplicates removed. All reviewers will be provided with the eligibility, inclusion and exclusion criteria, and a small subset of studies will be pilot tested. Two independent reviewers will screen the titles and abstracts of all the retrieved publications. Following this, two reviewers will independently screen the full texts to determine eligibility. Any disagreement between the two reviewers will be resolved by a third reviewer. Full details of the search results and study selections will be entered into a PRISMA flow diagram.

### Data extraction

Data extraction will be undertaken onto an Excel spreadsheet designed and agreed by the team members. The following details will be extracted:

- First author.
- Publication year.
- Type of study and characteristics of design.
- Location.
- Climate zone.
- Dates of study.
- Demographics (age, ethnicity, occupation, health status, body mass index, gestation age).
- Number of subjects.
- Inclusion/exclusion criteria.
- Definition and measurement of exposure/s.
- Definition and measurement of outcome/s.
- Gestational age at exposure assessment in relation to outcome.
- Main outcome.
- Confounders.
- Funding source.

If any of these details are missing from the selected studies, we will code these as ‘missing’ in the database. We anticipate that there will be differences in exposure and outcome definitions and measurements between the studies and will extract the full details of both of these to allow grouping of exposures or outcomes where possible in the analysis. If there are discrepancies in data extraction details, then a third reviewer will resolve the issues.

### Risk of bias analysis and quality assessment

Risk of bias will be assessed using the Office of Health Assessment and Translation (OHAT) tool. This includes evaluation of the following risks of bias in each study: selection, performance, detection/measurement, confounding, missing data and reporting. Each type of bias is given a score from low risk of bias to definitely high risk of bias. Methodological quality of the included studies will be assessed using the guidelines described by the OHAT in the ‘Handbook for Conducting a Literature-Based Health Assessment Using OHAT Approach for Systematic Review and Evidence Integration’. This will be performed by two independent reviewers and any disagreement will be resolved by moderation of a third reviewer. In studies with a high risk of bias and/or low quality, a decision will be made on whether to exclude the study. If the study remains in the systematic review, then conclusions based on this evidence will be caveated by the bias and/or quality assessment.

### Data synthesis

We will present summary statistics for each study included following the guidance on conducting a narrative synthesis of systematic reviews.^30^ If an outcome has been measured with at least two levels of exposure, we will conduct a dose–response meta-analysis if there are at least three studies with the same outcome. Should the outcome prevalence be below 10%, we will assume ORs are risk ratios to include in the meta-analysis. Where the prevalence is higher than 10%, we will transform the ORs to risk ratios. Meta-analysis on these will be conducted where at least three studies with the same outcome are included, to give an overall summary estimate of effect.

All meta-analyses will be conducted in R using the ‘metafor’ and ‘meta’ packages. Heterogeneity will be explored using the I^2^ statistic with CIs, which describes the proportion of variation due to heterogeneity rather than chance. We will also consider conducting a stratified analysis should there be significant study-level heterogeneity, to assess both within and between study variance, if there are enough studies to permit this.

### Subgroup analyses

We will consider several key subgroups to further our understanding of the different pathways that are important in evaluating the effect of environmental heat stress in pregnancy. Physical activity is unlikely to be an aspect of every study but is a key component of heat load and so we will summarise the difference between pregnant women exposed to environmental heat stress at rest versus those undergoing physical activity/work.

We will also summarise key differences and similarities in thermophysiology identified in studies with both pregnant and non-pregnant participants.

### Patient and public involvement

None.

### Ethics and dissemination

There was no need to seek ethical approval for this systematic review and meta-analysis. Dissemination will be through submission to a peer-reviewed journal, presentation to international conferences and to international interest groups.
