# Supplementary material for: Understanding the physiological and biological response to ambient heat exposure in pregnancy: protocol for a systematic review and meta-analysis
Source: BMJ Open. 2024 Jul 5;14(7):e085314. doi: 10.1136/bmjopen-2024-085314 (PMC11227802; doi:10.1136/bmjopen-2024-085314)
Supplement: Supplementary data [file bmjopen-2024-085314supp001.pdf]

Supplement for: Understanding the physiological and biological response to ambient heat exposure in pregnancy: a protocol for a systematic review and meta-analysis

Ana Bonell[1], Leonidas G Ioannou[2], Jane E Hirst[3,4], Andreas D Flouris[2]

1. Medical Research Council Unit The Gambia at London School of Hygiene and Tropical Medicine, Fajara, The Gambia
2. FAME Laboratory, Department of Physiocal Education and Sport Science, University of Thessaly, Thessaly, Greece
3. The George Institute for Global Health, School of Public Health, Imperial College London, London, UK.
4. Nuffield Department of Women's & Reproductive Health, University of Oxford, Oxford, UK.

Corresponding author: Ana Bonell, ana.bonell@lshtm.ac.uk

Bonell A, *et al. BMJ Open* 2024; 14:e085314. doi: 10.1136/bmjopen-2024-085314

Table 1: Gold standard papers to assess the search algorithm against

First Author	Year	Journal	Title
Smallcombe	2021	Sports Med	Thermoregulation during pregnancy: a controlled trial investigating the risk of maternal hyperthermia during exercise in the heat
Vaha-Eskeli	1991	Am J Perinatol	Doppler flow measurement of uterine and umbilical arteries in heat stress during late pregnancy
Bonell	2022	Lacet Planet Health	Environmental heat stress on maternal physiology and fetal blood flow in pregnancy subsistence farmers in The Gambia, west Africa: an observational cohort study
Wang	2020	Environ Int	Associations of maternal ambient temperature exposures during pregnancy with the placental weight, volume and PFR: a birth cohort study in Guangzhou, China
Shankar	2023	PNAS	Maternal nutritional status modifies heat-associated growth restriction in women with chronic malnutrition
Vaha-Eskeli	1991	Eur J Obstetrics & gynecology and reproductive biology	The effect of short-term heat stress on uterine contractility, fetal heart rate and fetal movements at late pregnancy
Abraham	2018	Environment International	Pregnancy exposure to atmospheric pollution and meteorological conditions and placental DNA methylation

Table 2: Search algorithm for Ovid Embase.

#	Search Algorithm – Ovid Embase 16/06/2024
1	(heat* and (stress or strain)).ti,ab.
2	(hot and (temperature* or condition* or environment* or workplace* or ambient*)).ti,ab.
3	(weather* or atmospher* or meteorolog*).ti,ab.
4	1 or 2 or 3
5	(maternal* or mother* or pregnan* or woman or women).ti,ab.
6	physiolog*.ti,ab.
7	heart rate*.ti,ab.
8	stroke volume*.ti,ab.
9	cardiac output*.ti,ab.
10	((skin* or core* or body or bodies) and temperature*).ti,ab.
11	(uterine* and blood flow*).ti,ab.
12	(hydrat* or euhydrat* or dehydrat* or hypohydrat*).ti,ab.
13	(vasomot* or vasoconstrict* or vasodilat*).ti,ab.
14	(sudomotor or sweat*).ti,ab.
15	(thermal and (comfort* or sensation*)).ti,ab.
16	(exertion or fatigue* or tired*).ti,ab.
17	(inflammat* and marker*).ti,ab.
18	heat shock protein*.ti,ab.
19	hormon*.ti,ab.
20	(plasma and glucose).ti,ab.
21	(placenta* and (epigenetic expression* or vasculature* or heat shock protein* or extracellular vesicle* or weight* or volume* or DNA methylation*)).ti,ab.
22	(umbilical and arter*).ti,ab.
23	((fetus* or fetal or antenatal or embryonic or prenatal or unborn) and (heart rate* or movement* or growth restriction* or (uterine and contraction*))).ti,ab.
24	6 or 7 or 8 or 9 or 10 or 11 or 12 or 13 or 14 or 15 or 16 or 17 or 18 or 19 or 20 or 21 or 22 or 23
25	4 and 5 and 24

Bonell A, *et al. BMJ Open* 2024; 14:e085314. doi: 10.1136/bmjopen-2024-085314
